# Genetic overlap between Alzheimer’s disease and immune-mediated diseases: An atlas of shared genetic determinants and biological convergence

**DOI:** 10.21203/rs.3.rs-3346282/v1

**Published:** 2023-09-28

**Authors:** Brisa Fernandes, Nitesh Enduru, Brisa Fernandes, Shahram Bahrami, Yulin Dai, Ole Andreassen, Zhongming Zhao

**Affiliations:** University of Texas Health Center at Houston; University of Texas Health Center at Houston; The University of Texas Health Science Center at Houston; The University of Texas Health Science Center at Houston; Oslo University Hospital & Institute of Clinical Medicine, University of Oslo; The University of Texas Health Science Center at Houston

**Keywords:** Alzheimer’s disease, immune-mediated diseases, genome-wide association studies, pleiotropy, Mendelian randomization, genetic correlation

## Abstract

The occurrence of immune disease comorbidities in Alzheimer’s disease (AD) has been observed in both epidemiological and molecular studies, suggesting a neuroinflammatory basis in AD. However, their shared genetic components have not been systematically studied. Here, we composed an atlas of the shared genetic associations between 11 immune-mediated diseases and AD by analyzing genome-wide association studies (GWAS) summary statistics. Our results unveiled a significant genetic overlap between AD and 11 individual immune-mediated diseases despite negligible genetic correlations, suggesting a complex shared genetic architecture distributed across the genome. The shared loci between AD and immune-mediated diseases implicated several genes, including *GRAMD1B, FUT2, ADAMTS4, HBEGF, WNT3, TSPAN14, DHODH, ABCB9* and *TNIP1*, all of which are protein-coding genes and thus potential drug targets. Top biological pathways enriched with these identified shared genes were related to the immune system and cell adhesion. In addition, *in silico* single-cell analyses showed enrichment of immune and brain cells, including neurons and microglia. In summary, our results suggest a genetic relationship between AD and the 11 immune-mediated diseases, pinpointing the existence of a shared however non-causal genetic basis. These identified protein-coding genes have the potential to serve as a novel path to therapeutic interventions for both AD and immune-mediated diseases and their comorbidities.

## Introduction

Alzheimer’s disease (AD) is a neurodegenerative brain disease that affects cognition, behavior and social skills ^1^. Twin studies have shown AD to be a highly heritable disease ^2^. As the older population is increasing, AD is becoming an emerging global public health challenge in our century, with an estimated 6.2 million Americans aged 65 and older living with AD. This number is projected to increase to 13.8 million by 2060^1^. An increasing body of evidence indicates that the pathogenesis of AD is not confined to the neurons but instead that neuroinflammation plays a significant role in the disease, with an interplay between the brain and the immune system^3^. In particular, the microglia, the brain-resident immune cells that contribute to the regulation of the brain immune response, is considered to have a pivotal role in the pathophysiology of AD, as a core alteration in AD^4,5^ or at least as a particular endophenotype in what is thought to be a heterogeneous disease^6^. Other studies pinpoint to alterations in peripheral immune cells in AD and a role for innate immune cells in the development of AD^7, 8^.

Chronic inflammation, which is often associated with immune-mediated diseases, has been implicated in the development and progression of AD. The comorbidities between immune diseases, showing immune system dysregulation, and AD have been widely reported. Epidemiological studies show that the overall incidence of AD is higher among individuals with inflammatory bowel disease, other inflammatory polyarthropathies and systematic connective tissue disorders, psoriasis, rheumatoid arthritis (RA), or multiple sclerosis compared to the age and sex-matched comparison groups free from inflammatory diseases^9–11^. A previous study investigated the pleiotropy between immune-mediated diseases and AD^12^. However, it is still unclear to what extent a multitude of immune-mediated diseases and AD share genetic underpinnings, and now larger and more powerful GWAS data are available.

In this study, we aimed to comprehensively investigate the genetic relationship, if any, between immune-mediated diseases and AD. We hypothesized that the genetic determinants contributing to AD overlap with those contributing to these immune-mediated diseases. To achieve this goal, we probed the shared genetic architecture between AD and a plethora of immune-mediated diseases. Specifically, we collected the genome-wide association studies (GWAS) summary statistics data from AD and 11 immune-mediated diseases: allergic rhinitis (AR), asthma, atopic dermatitis (AtD), celiac disease (CeD), Crohn’s disease (CD), hypothyroidism, primary sclerosing cholangitis (PSC), RA, systemic lupus erythematosus (SLE), ulcerative colitis (UC), and vitiligo. We identified the genes linked to the associated single nucleotide polymorphisms (SNPs) for each of the 11 immune-mediated diseases and AD. Moreover, we characterized the shared loci and their implications on genes, tissues, cell types, and biological functions between immune-mediated diseases and AD. Lastly, we ascertained their possible causal relationship, shedding light on the intricate interplay between these immune-mediated diseases and AD.

## Methods

### GWAS data sets used

For AD, we obtained GWAS summary statistics data from Wightman *et* al., which included only diagnosed AD individuals (n = 398,058)^13^. We used GWAS data with AD cases only, excluding AD proxies from UK Biobank, and such data was made available by the authors upon request. GWAS summary statistics data for AR (n = 289,307) ^14^, asthma (n = 385,822)^14^, atopic dermatitis (AtD) (n = 40,835)^15^, celiac disease (CeD) (n = 15,283)^16^, Crohn’s disease (CD) (n = 40,266)^17^, hypothyroidism (n = 244,890)^14^, primary sclerosing cholangitis (PSC) (n = 309,154)^18^, rheumatoid arthritis (RA) (n = 58,284)^19^, systemic lupus erythematosus (SLE) (n = 23,210)^20^, ulcerative colitis (UC) (n = 45,975)^17^, and vitiligo (n = 44,266)^21^ were obtained. Moreover, GWAS for hypertrophic cardiomyopathy (HCM)^22^ (n = 309,154) was obtained to serve as negative control for AD, considering that HCM is limited to heart pathology. All individuals were of European ancestry (EA). All summary statistics were built based on human reference genome GRCh37. There was no sample overlap between the AD GWAS and those immune-mediated diseases or the HCM GWAS datasets. Full details regarding the sample size of cases and controls for each GWAS is given in **Table S1**.

### Linkage disequilibrium score regression between AD and immune-mediated diseases

We calculated the genetic correlation between AD and 11 immune-mediated diseases using the cross-trait linkage disequilibrium score regression (LDSR) method^23^. Pre-estimated linkage disequilibrium (LD) scores provided by LDSC developers were obtained from the 1,000 Genomes Project (1kGP) European reference population; we preprocessed the summary statistics using LDSC munge_sumstats.py and used the odds ratio (OR) as the signed summary statistic. We calculated the genetic correlation by employing HapMap3 SNPs only with LD reference panel SNPs to minimize potential bias due to differences in LD structure.

### Genetic overlap between AD and immune-mediated diseases

To complement the genetic correlation analysis, we used the statistical tool MiXeR (https://github.com/precimed/mixer). MiXeR quantifies polygenic overlap irrespective of genetic correlation using GWAS summary statistics, and is agnostic to effect directions^24^. This method estimates the total number of both shared and trait-specific causal variants (i.e., variants with nonzero additive genetic effects on a given trait). We applied MiXeR to verify genetic overlap between AD and each immune-mediated disease. To evaluate model fit (the ability of the MiXeR model to predict the actual GWAS data), we constructed modeled vs. actual conditional quantile-quantile (Q–Q) plots, log-likelihood plots, and calculated the Akaike information criterion (AIC). A negative AIC value indicates that the MiXeR model cannot be adequately differentiated from a scenario of maximum possible overlap and a scenario of minimum overlap, while a positive AIC value indicates adequate fit for the MiXeR analyses. The major histocompatibility complex (MHC) region was excluded from the MiXeR analyses for all the considered traits.

### Conditional Q-Q plots to assess pleiotropic enrichment

We created conditional Q-Q plots to assess the cross-trait genetic enrichment, conditioning AD on the 11 immune-mediated diseases and one negative control, and we did this analysis inversely as well. The genetic enrichment can be referred to as a leftward shift in the Q-Q curve, indicating a considerable number of SNPs with their *p*-values greater than or equal to a given threshold. Q-Q plot is used to visualize the quantile distribution of the statistics for the observed result with respect to the expected quantile distribution under the null hypothesis, where the theoretical distribution is uniform on the interval [0,1]. In this case, enrichment is considered to occur when an increased proportion of SNPs associated with one phenotype or disease (AD) is observed as a function of the strength of the association with a second phenotype or disease, and enrichment is considered to occur in case the degree of the shift of the Q-Q curve to the left from the expected null line for the first disease is dependent on the magnitude of the association with the second disease^25^.

### Conditional and conjunctional false discovery rate analysis

Conditional false discovery rate (CondFDR) anlaysis was used to detect SNPs associated with AD given their association with each immune-mediated disease, and *vice versa*. CondFDR is defined as “the posterior probability that a given SNP is null (i.e., no SNPs associated with the first trait) for the primary trait (i.e., AD) given that the *p*-values for both traits are as small or smaller than the observed *p*-values”^25^. ConjFDR is defined as “the posterior probability that a given SNP is null for either phenotype or both simultaneously, given the *p*-values for both traits are as small or smaller than the observed *p*-values” ^25,26^. The enrichment verified in the conditional Q-Q plots is shown for each individual SNP according to the FDR. In this project, we used the conjFDR analysis with the threshold value 0.05 to identify the shared loci between AD and each of the 11 immune-mediated diseases. To examine the chromosomal location of SNPs in combination with AD and these immune-mediated diseases, we constructed a Manhattan plot using the bioinfokit package^27^.

### Local genetic correlation analysis to assess local genetic correlation

We used Local Analysis of covariant Association (LAVA) to further calculate the local genetic correlation between AD and immune-mediated diseases across the pleiotropic SNPs identified from conjFDR analysis. Using the pre-defined 2,495 genomic regions (2,492 after excluding the *APOE* region) provided by the LAVA developers, which minimize the local LD in those blocks^28^, the pleiotropic SNPs from conjFDR results were mapped and grouped into LAVA genomic regions. The genomic partition was performed using the 1kGP European individuals. To identify the presence of genetic signals, we initially performed a univariate test to estimate local heritability across each phenotype with a *p*-value < 0.05 being considered as a significant locus to evaluate for the bivariate local genetic correlation. For the loci with significant heritability in both disorders, we calculated the bivariate local genetic correlation. We adjusted the *p*-values using Bonferroni correction between AD and each immune-mediated disease based on the number of genomic loci tested in local univariate and bivariate analyses.

### Enrichment and assessment of SNP novelty

SNPs within the MHC region (defined as chr6: 25,119,106 – 33,854,733), the 8p23 inversion (chr8:7,200,000– 12,500,000), and apolipoprotein E (*APOE*) gene (chr19:44,000,000–47,000,000) were excluded from the analyses due to known association to AD^29, 30^ or a very complex LD structure ^31,32^ For the conjFDR analysis, we used a conjFDR *p*-value < 0.05 as statistically significant. A GWAS *p*-value _(AD & ID)_ > 5 x 10^−8^ cutoff in the original GWAS was used to identify the shared SNPs between two phenotypes as novel. In other words, to be considered ‘novel’, a SNP identified using the conjFDR should not have been found statistically significant in the original GWAS. For the local genetic correlation, we used a *p*-value < 0.05 as cutoff in the univariate and bivariate test with Bonferroni corrected *p*-value for AD and each immune-mediated disease. To check the novelty of the SNPs and loci identified in our study, we examined previously reported GWAS associations in the National Human Genome Research Institute (NHGRI)-EBI GWAS Catalog^33^. We further identified if the gene was novel to any of our traits based on the traits reported in the NHGRI-EBI GWAS Catalog^33^ and previous AD studies based on conjFDR analysis^25, 34–36^.

### Definition of genomic loci

Independent genomic loci were identified based on the FUMA protocol, an online tool for functional mapping of genetic variants (http://fuma.ctglab.nl/)^37^. SNPs with conjFDR < 0.1 were identified as independently significant and LD *r*^2^ < 0.6 with each other. A subset of these independent SNPs (LD *r*^2^ < 0.1) was then considered as the lead SNPs. The boundaries for genomic loci were then determined by identifying all candidate SNPs which were in LD (*r*^2^ ≥ 0.6) with the lead SNP Then, loci were combined if they were separated by less than 250 kb. These separate regions, containing all of these candidate SNPs, were treated as a single independent genomic locus. All LD information was calculated based on the EA reference panel from 1kGP^38^.

### Functional annotation and tissue and cell-specificity analyses

We annotated the genes to the lead SNPs based on positional mapping using ANNOtate VARiation (ANNOVAR) ^39^ in FUMA. We mapped all the candidate SNPs with conjFDR value < 0.1 and having an LD *r*^2^ ≥ 0.6 with one of the independently significant SNPs using ANNOVAR. In addition, these SNPs were annotated with Combined Annotation Dependent Depletion (CADD) scores ^40^ to predict how certain the SNP effect is on protein structure or function and possible contribution to genetic disease. Similarly, we used RegulomeDB (RDB version 1.1) scores ^41^ to predict the likelihood of regulatory functionality, and chromatin states, to predict transcription and also regulatory effects from chromatin states at the SNP locus. RegulomeDB (version 1.1) is a database that scores SNP’s functionality based upon its presence in a DNase hypersensitive site or transcription factor binding site. RDB ranks the SNPs with a score from 1 to 6. The SNP with strong evidence of being a regulatory variant is given the score of 1 and one with the least evidence is scored as 6^41^. SNPs with RDB scores of 1 (1a - 1f) were queried for known eQTLs in brain (GTEx v8, Braineac, CMC, PsychENCODE, xQTLServer), blood (GTEx v8, Blood eQTL, BIOS QTL, eQTLGen,) and immune system tissues (GTEx v8, DICE, scRNA eQTLs) along with other lead SNPs. An RDB score of 1 suggests the presence of the variant in multiple data regions such as combination of eQTL + TF binding + matched TF motif + matched DNase Footprint + DNase peak (for more details about the RDB score, see https://regulomedb.org/regulome-help/).

In addition, we ranked the tissues implicated by the SNPs using our in-house method DeepFun^42^. The DeepFun web server (https://bioinfo.uth.edu/deepfun/) is a convolutional neural network framework that was trained on ~ 8,000 chromatin profiles of 225 tissues or cell types from ENCODE and Roadmap projects. We imported all independent SNPs shared between AD and immune-mediated diseases to query the pretrained model on the web server and compute the SNP Activity Difference (SAD, ranging from − 1 to 1) between SNP alleles, indicating the accessibility or binding probability difference between reference and alternative alleles. We took the mean of absolute SAD of each SNP for each of the 50 unique tissues and cell lines and counted the number of tissues or cell types with the largest absolute SAD for each of the considered SNPs. The tissue and cell lines with most counts were defined as the most related tissue and cell lines to those independent SNPs shared between AD and immune-mediated diseases. We used all the shared SNPs between AD and immune-mediated diseases as the imputed SNPs. Then, we used Web-based Cell-type Specific Enrichment Analysis of Genes (WebCSEA, available at https://bioinfo.uth.edu/webcsea/)^43^ to assess the tissue and cell type enrichment of all the shared SNPs. WebCSEA curated a total of 111 single cell RNA-seq (scRNA-seq) panels of human tissues and 1,355 tissue-cell types (TCs) from 61 different general tissues across 11 human organ systems and uses decoding tissue-specificity (deTS) algorithm to measure the enrichment for each tissue-cell type^43^. In the WebCSEA analyses, a nominal *p*-value < 1 x 10^−3^ was considered statistically significant.

Finally, we used FUMA to analyse the Gene Ontology (GO) terms in three domains (biological processes, molecular functions, and cellular components terms) and biological pathways from KEGG (Kyoto encyclopedia of genes and genomes), Panther BD, Reactome, and Wikipathways. An FDR corrected by Benjamini and Hochberg (BH) procedure^44^ < 0.05 was considered significant for the GO and pathway analyses.

### Causal relationship between AD and immune-mediated diseases

We evaluated the potential causal relationship between AD and immune-mediated diseases using Mendelian randomization (MR) analysis. MR uses SNPs as instrument variables (IV) to assess causality and these IVs are defined by three assumptions^45^. First, the selected IVs are significantly associated (*p*-value_GWAS_ < 5 x 10^−8^) with the exposure variable. Second, the IVs are independent between the exposure and the outcome. Third, the effect of IVs on the outcome must precede through the exposure. We used the two-sample MR (2SMR) method (https://mrcieu.github.io/TwoSampleMR/articles/introduction.html). Initially, independent (*r*^2^ < 0.001) genome-wide significant SNPs (*p*-value_GWAS_ < 5 × 10^−8^) associated with the exposure (immune-mediated diseases) were considered as IVs and assessed against outcome variables (AD). We used the five MR methods [MR Egger, Weighted median, Inverse variance weighted (IVW), Simple mode, and Weighted mode] implemented in the 2SMR *R* package^46^.

## Results

**Figure 1:**
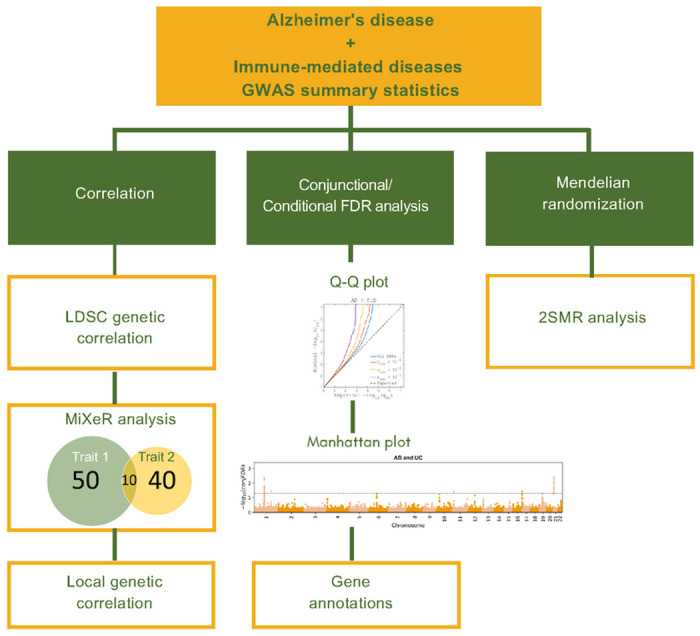
Workflow for the study of AD and 11 immune-mediated diseases.

The general workflow of our study is illustrated in Fig. 1. We developed a pipeline to perform the global and local genetic correlations between AD and 11 immune-mediated diseases and to quantify the shared loci among them using MiXeR. We used the conditional/comjunctional FDR to identify the shared loci and, finally, performed bi-directional MR to assess the causal relationship between AD and the 11 immune-mediated diseases.

### Genetic correlation and genetic overlap beyond genetic correlation

We computed the genetic correlation based on the LDSR method. In general, there was no significant genetic correlation between AD and AR (*r*_*g*_ = 0.043, standard error (SE) = 0.08, *p* = 0.6), AD and asthma (*r*_*g*_ = 0.005, SE = 0.06, *p* = 0.9), AD and AtD (*r*_*g*_ = −0.023, SE = 0.11, *p* = 0.8), AD and CeD (*r*_*g*_ = −0.011, SE = 0.12, *p* = 0.9), AD and CD (*r*_*g*_ = 0.0006, SE = 0.02, *p* = 0.9), AD and hypothyroidism (*r*_*g*_ = 0.05, SE = 0.07, *p* = 0.5), AD and PSC (rg = −0.09, p = 0.2) AD and RA (*r*_*g*_ = −0.12, SE = 0.07, *p* = 0.1), AD and SLE (*r*_*g*_ = −0.05, SE = 0.1, *p* = 0.6), AD and UC (*r*_*g*_ = 0.03, SE = 0.02, *p* = 0.2), and AD and vitiligo (*r*_*g*_ = 0.03, SE = 0.08, *p* = 0.7). In addition, no genetic correlation was found between AD and the negative control HCM (*r*_*g*_ = 0.14, SE = 0.18, *p* = 0.42) (**Table S2**).

**Figure 2:**
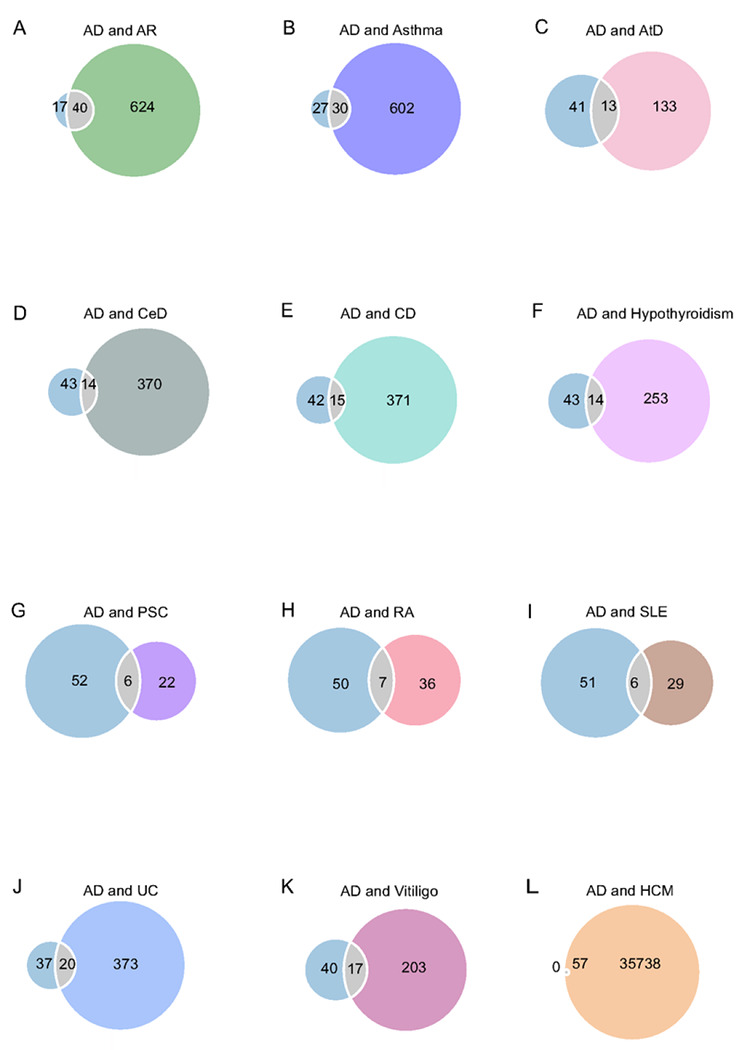
Genetic overlap between AD and 11 immune-mediated diseases using MiXeR. Venn diagrams represent the genetic overlap, regardless of direction, between AD and each immune-mediated disease. The size of the circles represents the polygenicity of the phenotype. Numeric values represent the estimated number of variants. Abbreviations: AD: Alzheimer’s disease; AR: allergic rhinitis; AtD: atopic dermatitis; CeD: celiac disease; CD: Crohn’s disease; HCM: hypertrophic cardiomyopathy; PSC: primary sclerosing cholangitis; RA: rheumatoid arthritis; SLE: systemic lupus erythematosus; and UC: ulcerative colitis.

To capture mixtures of effect directions across shared genetic variants, we performed MiXeR analysis with AD and immune-mediated diseases to verify genetic overlap beyond genetic correlation by determining the number of overlapping variants between trait pairs. The observed small genetic overlap in the Venn diagrams with nonexistent genetic correlations suggests a balanced mixture of concordant and discordant genetic effects across shared loci between AD and immune-mediated diseases, with adequate quality of model fit as suggested by positive AIC values. The predicted number of shared causal loci between AD and immune-mediated diseases varied from 6 (between AD and PSC; AD and SLE) to 40 (AD and AR). There was complete genetic overlap between AD and the negative control HCM. However, the AIC values were all negative, indicating a poor model fit and, thus, this particular analysis is unreliable (Fig. 2, **Figure S1**, and **Table S3**).

### Pleiotropic enrichment

**Figure 3:**
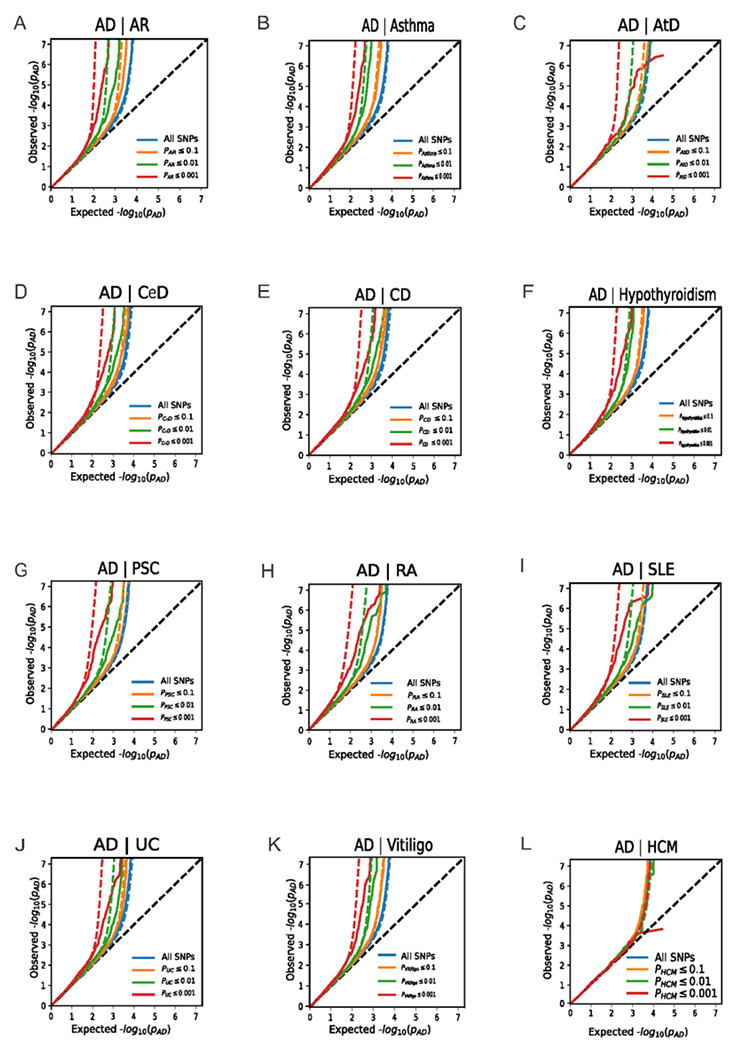
Conditional quantile-quantile plots of nominal vs. empirical −log_10_ *p*-values in AD below the standard genome-wide association study threshold of *p*-value < 5 x 10^−8^ as a function of significance of association with immune-mediated diseases at the level of −log_10_ *p*-values of 1, 2, or 3, corresponding to *p*-value = 0.1, *p*-value = 0.01, and *p*-value = 0.001, respectively. Significant association was found between AD and all immune-mediated diseases. No significant association was found between AD and HCM (negative control). The null hypothesis is indicated by the dashed lines. Abbreviations: AD: Alzheimer’s disease; AR: allergic rhinitis; AtD: atopic dermatitis; CeD: celiac disease; CD: Crohn’s disease; HCM: hypertrophic cardiomyopathy; PSC: primary sclerosing cholangitis; RA: rheumatoid arthritis; SLE: systemic lupus erythematosus; and UC: ulcerative colitis.

The enrichment observed in QQ-plots can be translated to FDR for each SNP The genetic enrichment can be identified from the Q-Q plot with a leftward shift in the Q-Q curve, indicating a considerable number of SNPs with *p*-value greater than or equal to a given threshold. There was an enrichment of associations with AD given increasing SNP associations with each immune-mediated disease and conversely in the conditional Q-Q-plots, with a leftward shift of decreasing values of empirical − log10(q_AD_), showing polygenic overlap between AD and each immune-mediated disease across common genetic variants. No enrichment was verified between AD and the negative control HCM (Fig. 3, **Figure S1**).

### Shared and novel loci for AD and immune-mediated diseases

We employed conjFDR to ascertain SNPs mutually associated with AD and each immune-mediated disease; in order to determine the allelic direction of effects in the diseases, we used the *z*-scores from the original GWAS. Considering a conjFDR < 0.05, we identified 76 shared genomic loci (**Table S4**), of which 6 loci were jointly associated with more than one pair of diseases. Five of the 6 multi-trait shared loci were identified as novel loci for AD (**Table S5**). After excluding these 6 overlapping SNPs, we identified 70 unique shared genomic loci (71 unique SNPs) between AD and immune-mediated diseases, out of which 38 genomic loci were novel for AD only and 25 genomic loci were novel for immune-mediated diseases only (Tables 1 and S4); 17 genomic loci were identified as novel to both AD and immune-mediated diseases (Table 2). We then constructed conjFDR Manhattan plots to visualize all the novel and all shared loci between these diseases (**Figure S2**), and, based on the *z*-scores, we evaluated the directionality of allelic effects in the loci shared between AD and the immune-mediated diseases. All the shared loci had a mixed direction of effect, except for AtD and AD, which had complete concordance effect (Tables 1 and S4). The shared loci (1q23.3, 7p15.1, 22q13.2, 1q32.1, 4p14, 6q23.3) and their respective genes (*ADAMTS4, JAZF1, Y_RNA, C1orf106, AC195454.1*, and *RP11-95M15.1*) were mapped to more than one immune-mediated disease. No shared loci were identified between AD and the negative control HCM (**Figures S2** and **4** and **Table S5**).

### Local genetic correlation analysis across the LAVA predefined genomic loci and conjFDR results

Regional genetic correlations may be masked when *r*_*g*_ is assessed on a genome-wide level. To investigate whether there are any genomic loci with pronounced genetic correlations despite negligible genome-wide *r*_*g*_ we estimated regional genetic correlations using LAVA. Regional genetic correlations are a better tool to capture genetic associations with mixed effect directions. That is, a pair of traits may exhibit no global genetic correlations as a result of an equal number of positive and negative (opposite effect directions) local genetic correlations of similar magnitude. We initially performed LAVA on the pre-defined 2,492 genmic loci to calculate the pairwise local genetic correlation across AD and 11 immune-mediated diseases. We used a nominal *p*-value < 0.05 to detect the univariate signals and used the Bonferroni corrected *p*-value between AD and each of the 11 immune-mediated diseases for bivariate analysis. The LAVA results were summarized in **Table S6**. The overall average concordant effect in univariate analysis was 7%, and 46% of these loci had 95% confidence intervals (CIs) for the variance that included 1. The overall average concordant effect in bivariate analysis was 53%, and 81% of these loci had 95% CIs for the variance that included 1. The overall average concordant effect in Bonferroni adjusted bivariate analysis was 56%, and 87% of these loci had 95% CIs for the variance that included 1. Overall 1,765 unique loci found to univariately heritable (**Table S8**) and 745 unique loci found significant for bivariate analysis at *p*-value < 0.05 for AD and 11 immune-mediated diseases (**Table S9**) and 220 unique loci are shared between more than one immune-mediated disease with AD (**Table S10**). After adjusting for Bonferroni correction between AD and each of the 11 immune-mediated disease, we found 39 genomic loci shared between AD and 11 immune-mediated diseases (**Table S11**).

Based on the conjFDR analysis results, we further performed local genetic correlation using LAVA on the 17 novel and 59 previously reported shared loci (6 of these loci are shared between multiple pair of traits, see **Table S5**) (**Tables S4 and S7**) identified between AD and immune-mediated diseases. Among the 17 novel loci, 11 were significantly heritable at univariate *p*-value < 0.05, and 3 were bivariate significant at *p*-value < 0.05 and 5 of those loci had 95% confidence intervals (CIs) for the variance that included 1 (**Table S12**). After adjusting for Bonferroni correction, 2 out of 3 loci were statistically significant for correlation. Thirty-four of the 59 loci had significant heritability for both diseases, and 7 of those loci had a bivariate *p*-value < 0.05; 7 of those loci had 95% CIs for the variance that included 1, suggesting a shared signal between the diseases and that the local genetic signal of those phenotypes is completely shared even though they are not statistically significant (**Tables S7 and S12**). After adjustment using Bonferroni correction, 6 out of 7 loci were statistically significant for correlation. Overall, 8 out of 76 loci had a Bonferroni adjusted bivariate *p*-value < 0.05. These results provide further evidence of pleiotropy with mixed effect direction between AD and immune-mediated diseases with 3 positively correlated and 5 negatively correlated at Bonferroni adjusted bivariate *p*-value < 0.05. We found only one genomic locus (chr 2:64696090–65938002) shared between LAVA and ConjFDR results.

### Exploration of shared biological mechanisms: Functional annotation and tissue and cell-specificity analyses

Functional annotation for all the lead SNPs at conjFDR < 0.05 within the loci shared between AD and immune-mediated diseases showed that the majority of the lead SNPs are intronic or intergenic (**Table S4**). Two lead SNPs were reported to have a CADD score > 12.37, suggesting deleteriousness (rs12790721 and rs602662, *GRAMD1B* and *FUT2*, respectively) ^40^. Seven lead SNPs reported a low RegulomeDB scores of 1d or 1f, suggesting regulatory functionality (rs4233366, rs739954, rs2074404, rs10748526, rs3764310, rs4275659 and rs4958435, nearest genes *ADAMTS4, HBEGF, WNT3, TSPAN14, DHODH, ABCB9 and TNIP1*, respectively)^41^ (**Table S13**). These SNPs were associated with eQTL functionality in different brain, blood, and immune system regions. For a few examples, we found eQTL functionality for genes *NDUFS2, PCP4L, PCDHA8, SRA1, PCDHA10, VTRNA1-3, PCDHA3, PCDHA6, PCDHB15, WDR55, CYSTM1* and 739 unique genes (**Table S14**). Functional annotation for all SNPs associated with lead SNPs at conjFDR < 0.1 and *r*^2^ > 0.6 were extracted from GWAS summary statistics, between AD and immune-mediated diseases. The majority of the candidate SNPs are either intronic or intergenic (**Table S15**). In **Figures S2 and 4**, the shared genes between AD and each specific immune-mediated disease are shown.

Functional annotation of the lead SNPs using positional, eQTL and chromatin interaction mapping was performed to understand the biological mechanism affecting AD and immune-mediated diseases. Based on the three gene mapping techniques, we identified 142 unique credible genes mapped to the lead SNPs between AD and immune-mediated diseases (**Table S16**). The credible genes were mapped to the selected eQTLs available in FUMA. Out of the 70 unique shared loci (71 unique SNPs) identified using the conjFDR method, 50 loci were identified as eQTLs covering 750 unique genes across the different brain, blood, and immune system tissues in the GTEx database (**Table S16**).

To understand the tissues that these SNP loci tend to manifest their impacts, we leveraged the in-house method DeepFun and identified that three out of the top-ranked five tissues were immune and brain tissues (thymus, cerebellum, and frontal cortex) (**Figure S3**). We mapped these SNP loci to the proximity gene using the online tool WebCSEA, and found enrichment in the lymphatic system. There was a signal in the nervous, cardiovascular, and endocrine systems (**Figure S4**); the top enriched cell types were mostly related to the immune and nervous systems (neutrophil, macrophage, monocytes, innate lymphoid cell, plasma cell, excitatory neuron, purkinje cell, microglia, dendritic and glial cells) (**Figure S5**) (nominal significance *p*-value < 1 x 10^−3^). Finally, the top biological pathways were related to cell adhesion and the immune system (**Table S17**).

### Lack of significant causal association observed between AD and immune-mediated diseases

We evaluated the potential causal relationship between immune-mediated diseases and AD using five MR methods (MR Egger, weighted median, inverse variance weighted (IVW), simple mode, and weighted mode) implemented in the 2SMR package. There was no sufficient evidence for causal relationship between immune-mediated diseases and AD in all these tests, except for asthma which had a significant causal relationship with AD based on weighted median and weighted mode methods (**Table S18**).

## Discussion

In this study, we performed a comprehensive assessment of the shared genetic architecture between AD and immune-mediated diseases by analyzing large-scale GWAS summary data using different but complementary genetic approaches in order to shed light into their shared underlying molecular biology mechanisms. The findings show extensive genetic overlap between AD and immune-mediated diseases regardless of genetic correlation. We identified several shared and novel loci using the conjFDR approach and identified several pathways and immunological signatures enriched in the brain and immune system.

In our analyses, LDSR did not show significant genome-wide genetic correlation between AD and each immune-mediated trait. Still, the evidence of significant genetic overlap verified by MiXeR in tandem with nonsignificant genetic correlation reflects shared genetic etiologies with mixed effect directions, a suggestion which is corroborated by the local genetic correlation performed using LAVA; this balanced mixture of concordant and discordant shared loci distributed across the genome indicates that some genetic variants can increase the risk of one disorder while decreasing the risk of the other. In addition, all MR analyses, with the exception of perhaps asthma, gave us no support for a significant causal relationship between AD and immune-mediated diseases, indicating that pleiotropic and common biological pathways may be a better explanation for their association. A similar study showed no significant causal relationship between AD and immune-mediated diseases with the exception of multiple sclerosis and Sjogren’s syndrome^47^. In our study, a total of 70 unique shared genomic loci were identified between AD and immune-mediated diseases by the conjFDR analyses employing GWAS data, being enriched in biological pathways related to cell adhesion and the immune system. They were mostly enriched in the lymphatic system, with a signal being seem in the central nervous system; the top enriched cell types were also related to the immune and nervous systems, including neurons and microglia.

Two SNPs were suggestive of deleteriousness, rs12790721 and rs646327. The first is an intron variant and eQTL of *GRAMD1B* (11q24.1) in blood, and the second is an exonic variant of *FUT2* (19q13.33 ). Both genes are protein-coding genes, for the proteins aster-B and galactoside alpha-(1, 2)-fucosyltransferase 2, respectively. *GRAMD1B* is expressed in the brain, in astrocytes, oligodendrocytes precursor cells, and in inhibitory and excitatory neurons, mainly maintaining synaptic function, and is also expressed in the immune system. It is predicted to be involved in cellular response to cholesterol and cholesterol homeostasis. Aster-B, as a novel regulator of mitochondrial cholesterol and fatty acid transport, and excess mitochondrial cholesterol, has also been a biomarker in AD. Because abnormal mitochondrial cholesterol is a common phenotype in AD, Aster-B has been suggested as a target for the development of therapeutics for AD^48^. Regarding *FUT2*, fucosylated host glycoproteins or glycolipids mediate interaction with intestinal microbiota, influencing its composition^49–51^. There is evidence suggesting a link between gut microbiota dysbiosis and the pathogenesis of AD, with the gut microbiota modulating neuroinflammation indirectly by impacting microglia and having effects on synaptic neurotransmission dysfunction, with a cross-talk between peripheral and central inflammation through microbiota-mediated microglial alterations^52, 53^.

In addition to deleteriousness, seven lead SNPs were considered to have important regulatory functionality, mapped to the genes *ADAMTS4* (UTR3 region), *HBEGF*(intergenic), *WNT3* (intronic), *TSPAN14* (intronic), *DHODH* (intronic), *ABCB9* (intronic) *and TNIP1* (intronic), all of each are protein-coding genes. For instance, the protein coded by *ADAMTS4* is expressed in the cytoplasm in several tissues, being most abundant in the central nervous system. Its RNA is mostly expressed in oligodendrocytes, and is related to myelination. The enzyme coded by *ADAMTS4* is responsible for amyloid deposition in AD^54, 55^. *HBEGF* has been associated with AD, with its overexpression resulting in increased APP protein level^56^. It is expressed in the cortex and hippocampus, mostly in neurons and astrocytes, and also in the immune system. Genetic deletion of *HBEGF* cause cognitive dysfunction, which is reversed by NMDA receptor agonists^57^. Heparin-binding epidermal growth factor-like growth factor (HB-EGF), coded by *HBEGF*, also restores neurogenesis in the hippocampus of aged mice^58^. It also contributes to the proliferation of glial cells and to the survival of dopaminergic neurons^59^. *DHODH*, it is detected in the immune system and is related to mitochondrial function and cellular homeostasis and also to alterations in reactive oxygen species levels^60^. *TNIP1* is related to inflammatory response, including neuroinflammation and microglia activation, in addition to regulating nuclear factor kappa-B activation^61^, being related to AD^62^. Finally, in the loci to eQTL analyses, several SNPs were found associated with eQTL functionality in brain tissue and in the immune system. For instance, *NDUFS2* gene is a protein-coding gene that is associated with mitochondrial complex I alterations. A transcriptome-wide association study of AD using brain tissue found that *NDUFS2* is one of the putative causal genes in AD^63, 64^.

Six genes were found to be shared between AD and at least two immune-related diseases: *ADAMTS4, JAZF1, Y_RNA, C1orf106, AC195454.1*, and *RP11-95M15.1*. As discussed above, *ADAMTS4* is responsible for amyloid deposition in AD^54, 55^. *JAZF1* is a protein-coding gene which functions as a transcriptional repressor; it is involved in glucose and lipid metabolisms^65^. *Y_RNA* is a small non-coding RNA gene. It has been implicated in cellular processes such as DNA replication and RNA quality control. Y_RNA has been found in extracellular vesicles (EV) from multiple different cell lines, and EV-associated Y-RNA may be involved in a range of immune-related processes, including inflammation and immune suppression, being regulated in immune cells by Toll-like receptor (TLR) signaling^66^. Dysregulation of Y-RNA also has been found to cause alternative splicing in neurons of individuals with AD^67^. *C1orf106*, also known as *CALHM6*, encodes the protein calcium homeostasis modulator, which may play an important role in several immune inflammatory responses. It is upregulated by interferon-gamma and tumor necrosis factor alpha^68^. *AC195454.1* is a long non-coding gene with unknown functional category. It is overexpressed in the brain and has been implicated in SLE^69, 70^. Finally, *RP11-95M15.1*, also a long non-coding gene, has been associated with PSC^71^.

One of the major strengths of our study is the employment of diverse albeit complementary statistical genetic approaches, enabling an extensive analysis of the genetic associations between AD and immune-mediated diseases. In addition, the fact that we included generally well-powered GWAS suggests that our results are mostly not due to small sample sizes. Another strength is that we included only diagnosed cases of AD and not by proxy subjects. Having said that, our study has inherent limitations that should be considered alongside the present findings. The analyses were restricted to participants of European ancestry; thus, our findings may not be generalizable to populations of other ancestries. In addition, since we used the most comprehensive GWAS data available, replication analyses in independent samples were not possible. Also, AD is not a highly polygenic disease, which might have underestimate the number of variants found in the MiXeR analyses. Further, the AIC values from the MiXeR analysis for the negative control HCM were all negative, showing poor fit and that the results of this particular analysis are unreliable. Finally, the gene mapping strategy was based on statistical analyses, and should be validated with experimental studies.

In summary, our study provides genetic insights into the observed epidemiological relationship between AD and immune-mediated diseases, exposing shared genetic susceptibility extensively distributed across the genome. Our results support a significant genetic association between AD and immune-mediated diseases with mixed effect directions, with some genes increasing the risk of one disease but decreasing the risk of the other. Furthermore, we pinpoint shared loci and genes between AD and immune-mediated diseases that have the potential to be intended for further research, such as *GRAMD1B, FUT2, ADAMTS4, HBEGF, WNT3, TSPAN14, DHODH, ABCB9 and TNIP1*, all of which are protein-coding, and thus show promise as potential drug targets, and showed deleteriousness or regulatory functionality. We also highlight the significance of the immune system as a shared mechanism, albeit a non-causal one, in AD and immune-mediated diseases, providing further support for the immunoinflammatory hypothesis in AD. Overall, our results provide an atlas of the shared genetic architecture of AD and immune-mediated diseases, and their long-noticed comorbidity association, with implications for personalized interventions for the prevention and treatment of AD and immune-mediated diseases.

## Figures and Tables

**Figure 4:**
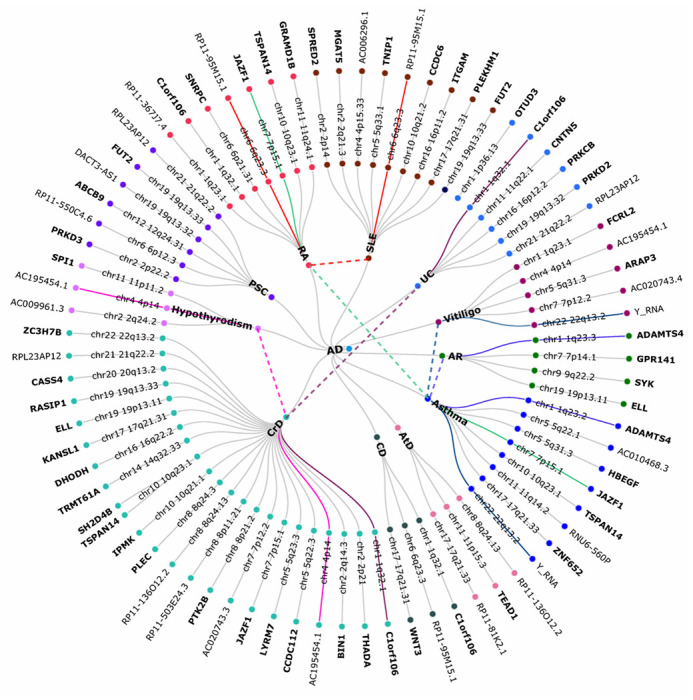
A circular dendrogram showing the shared genes between AD (center circle) and each of eleven specific immune-mediated diseases (first circle), resulting in eleven pairs. A total of 70 unique shared loci were identified across eleven trait pairs, which mapped to 43 distinct protein-coding genes and 16 distinct non-protein coding genes (second and third circles) identified using ANNOVAR. The dashed lines denote genes shared between AD and more than one immune-mediated disease. Genes in bold font suggest protein-coding genes. Abbreviations: ANNOVAR: analysis ANNOtate VARiation (ANNOVAR); AD: Alzheimer’s disease; AR: allergic rhinitis; AtD: atopic dermatitis; CeD: celiac disease; CD: Crohn’s disease; HCM: hypertrophic cardiomyopathy; PSC: primary sclerosing cholangitis; RA: rheumatoid arthritis; SLE: systemic lupus erythematosus; and UC: ulcerative colitis.

**Table 1:** Summary of total loci shared between Alzheimer’s disease (AD) and immune-mediated diseases (ID).

Immune-mediated disease (ID)	# loci	# novel AD loci	Concordance effect (%) for AD novel loci	# novel ID loci	Concordance effect (%) for novel ID loci	# novel loci for AD & ID	Concordance effect (%) for novel loci
Allergic rhinitis	4	3	66.7	2	100	2	100
Asthma	8	2	50	3	33.3	1	100
Atopic dermatitis	3	1	100	2	100	1	100
Celiac disease	3	1	100	1	100	0	NA
Crohn’s disease	22	11	36.4	6	16.7	5	20
Hypothyroidism	3	2	50	0	0	0	NA
Primary sclerosing cholangitis	6	2	50	3	66.7	0	NA
Rheumatoid arthritis	7	4	25	1	100	1	100
Systemic lupus erythematosus	9	5	20	3	33.3	3	33.3
Ulcerative colitis	6	4	50	3	33.3	3	33.3
Vitiligo	5	3	0	1	0	1	0
Overall	76	38	49.8	25	53	17	60.8

AD = Alzheimer’s disease. ID = immune-mediated disease.

**Table 2:** Novel loci associated with AD and Immune-mediated diseases based on conjunctional false discovery rate analysis.

Trait1	Trait2	Locus	Chr	SNP	Pos	Nearest Gene	Distance	Function	A1/A2	zscore_Trait1	zscore_Trait2	conjfdr_Trait1_Trait2	P_Trait1	P_Trait2
AD	AR	1	7	rs17171172	37872650	GPR141, EPDR1*	0:00	intronic	T/C	3.9	4.1	1.92E-02	9.86E-05	3.46E-05
AD	AR	2	9	rs10993704	93597359	SYK	0	intronic	T/C	3.7	3.8	4.75E-02	2.11E-04	1.38E-04
AD	Asthma	3	5	rs2028258	110553497	AC010468.3	8451	intergenic	A/G	4.3	3.9	2.43E-02	1.58E-05	9.53E-05
AD	AtD	4	8	rs897150	126620703	RP11-136O12.2	23086	intergenic	A/G	4.3	4.4	8.55E-03	1.90E-05	1.05E-05
AD	CD	5	5	rs2963769	114632469	CCDC112*	0	intronic	T/C	3.7	−3.8	3.64E-02	2.23E-04	1.38E-04
AD	CD	6	8	rs2923447	42457590	RP11-503E24.3	29511	intergenic	T/G	3.6	−3.7	4.70E-02	3.19E-04	2.52E-04
AD	CD	7	10	rs9665686	82302047	SH2DB4	0	Intronic	T/C	3.7	3.4	4.74E-02	2.73E-04	2.80E-04
AD	CD	8	14	rs45441198	104010198	TRMT61A	0	UTR3	T/C	4.0	−4.8	1.33E-02	5.78E-05	1.56E-06
AD	CD	9	16	rs3764310	72073122	DHODH	0	intronic	T/C	−3.8	4.6	2.76E-02	1.53E-04	3.56E-06
AD	RA	10	11	rs12790721	123385425	LINC01059	12142	intergenic	T/C	3.8	3.5	4.39E-02	1.65E-04	4.30E-04
AD	SLE	11	2	rs62168048	135152287	MGAT5**^**	0	intronic	A/G	−4.1	4.1	1.28E-02	3.67E-05	3.61E-05
AD	SLE	12	4	rs16890310	14378658	AC006296.1	17952	intergenic	A/G	−3.7	4.1	4.67E-02	2.57E-04	3.77E-05
AD	SLE	13	10	rs4948365	61574694	CCDC6	0	intronic	T/C	3.7	3.8	4.24E-02	1.97E-04	1.55E-04
AD	UC	14	1	rs148784768	20242739	OTUD3**^**	22669	intergenic	A/G	3.9	−5.1	3.74E-02	1.17E-04	2.76E-07
AD	UC	15	11	rs117197442	100142344	CNTN5	0	intronic	T/C	−3.8	4.2	3.90E-02	1.24E-04	2.32E-05
AD	UC	16	16	rs11644964	23962293	PRKCB**^**	0	intronic	A/G	3.9	4.2	3.60E-02	1.12E-04	2.40E-05
AD	Vitiligo	17	5	rs62380789	141050575	ARAP3	0	intronic	A/C	4.0	−4.4	2.57E-02	8.11E-05	3.08E-05
